# Development of Bovine-Derived *β-Actin* Genomic DNA Reference Materials for Meat Adulteration

**DOI:** 10.3390/ijms27198623

**Published:** 2026-09-26

**Authors:** Piao Feng, Yi Ji, Cheng Peng, Lei Yang, Xiaofu Wang, Liang Li, Tao Huang, Junfeng Xu, Xiaoyun Chen

**Affiliations:** 1 State Key Laboratory for Quality and Safety of Agro-Products, College of Food Science and Engineering, Ningbo University, Ningbo 315211, China; tk1236911@163.com; 2 State Key Laboratory for Quality and Safety of Agro-Products, Key Laboratory of Traceability for Agricultural Genetically Modified Organisms, Ministry of Agriculture and Rural Affairs, Zhejiang Key Laboratory of Crop Germplasm Innovation and Utilization, Zhejiang Academy of Agricultural Sciences, Hangzhou 310021, China; jymemory12138@163.com (Y.J.); pc_phm@163.com (C.P.); yanglei9203@163.com (L.Y.); ywxf1981@163.com (X.W.); njifxu@163.com (J.X.); 3 Institute of Quality Standard and Testing Technology for Agro-Products, Chinese Academy of Agricultural Sciences, Beijing 100081, China; liliang@caas.cn

**Keywords:** food adulteration, reference materials, bovine-derived *β-actin* gene, droplet digital PCR

## Abstract

Meat quality and authenticity are critical food safety issues, especially for processed meat products prone to species mislabeling and adulteration. Accurate molecular identification of beef relies on species-specific DNA markers, and nucleic acid reference materials (RMs) serve as the “yardstick” in molecular metrology. However, the lack of single-copy gene DNA RMs has limited detection accuracy and reliability. To address this, we developed and validated certified reference materials (CRMs) for identifying beef in food products using bovine genomic DNA. The *β-actin* gene in beef was detected by digital PCR to establish these CRMs. The certified values of *β-actin* in the CRMs were determined to be (6.85 ± 0.63) × 10^3^ copies/μL and (8.00 ± 0.77) × 10^2^ copies/μL, with extended uncertainty (coverage factor k = 2), based on *β-actin* quantification by 9 independent collaborating laboratories. Furthermore, these CRMs were used to evaluate the standard suitability, analyze quantitative deviations between single-copy gene and multi-gene approaches, and test with real samples. These efforts provide robust support for expanding the national catalog of animal food ingredient CRMs and enhancing detection accuracy.

## 1. Introduction

Meat products constitute a vital component of the human diet, serving as a primary source of high-quality protein and fat while also representing one of the main vehicles for obtaining micronutrients and minerals [1]. However, food adulteration and fraud remain severe challenges in global food safety, threatening consumer health, undermining consumer rights, and eroding public trust in the food system [2]. Food fraud manifests in diverse forms, including adulteration, substitution, dilution, tampering, simulation, counterfeiting, and misrepresentation [3]. A survey of 1,553 food fraud cases in China revealed that approximately 10% involved meat and meat products [4], identifying this category as a high-risk target. Documented incidents include adulterated beef balls [5] and mutton products [6,7]. Beyond economic fraud, accurate species identification carries profound legal and cultural dimensions: in countries such as India, Nepal, and Japan, consumption of beef and beef products is explicitly restricted by religious, legal, and cultural traditions. Accurate identification of meat species is therefore indispensable for safeguarding consumers’ right to know, preventing food fraud, upholding religious beliefs, and mitigating public health and safety risks.

Nucleic acid-based detection methods have become the cornerstone of food authenticity testing, leveraging interspecies sequence divergence as a molecular fingerprint. Fluorescent quantitative PCR (qPCR) is widely employed for qualitative and relative quantitative detection in meat products [8,9,10]. However, qPCR suffers from well-documented limitations: sensitivity to PCR inhibitors present in complex food matrices, limited resolution for trace-level adulteration, and reliance on external standard curves that introduce inter-laboratory variability [11,12]. Digital PCR (dPCR), first proposed at the end of the 20th century [13], addresses these shortcomings through limiting-dilution partitioning and end-point fluorescence measurement, enabling absolute quantification without external calibrators. dPCR has demonstrated superior tolerance to inhibitors [14,15], enhanced sensitivity for low-abundance targets, and excellent inter-laboratory reproducibility, positioning it as a candidate reference measurement procedure for nucleic acid quantification.

Despite advances in measurement technology, a critical bottleneck persists: the comparability of test results across different laboratories is severely compromised by the absence of unified reference materials (RMs) and standardized protocols for meat species quantification. Nucleic acid reference materials (NARMs), valued for their demonstrated homogeneity and stability, serve as metrological anchors that link measurements to a common reference point [16]. They have been successfully deployed in medical diagnostics, food testing, and veterinary disease control [17]. In the specific context of bovine-derived ingredient quantification, most existing detection standards (e.g., SN/T 2557-2010 [18], GB/T 25165-2010 [19], GB/T 38164-2019 [20]) target multi-copy mitochondrial or ribosomal genes (cytb, GH, 18S rRNA) to maximize detection sensitivity. These alphanumeric codes denote Chinese standard document numbers and their publication years (e.g., SN/T = entry-exit inspection and quarantine industry standard; GB/T = national recommended standard; the final four digits indicate the year of issue). However, multi-copy genes exhibit variable copy numbers across tissues, breeds, and physiological states, rendering them inherently unreliable as quantitative targets: the measured gene copy number does not correspond to a fixed number of genome equivalents, precluding accurate calculation of adulteration levels on a mass or genome basis. Single-copy nuclear genes, by contrast, provide a stoichiometric 1:1 relationship between gene copies and genome copies, making them the metrologically sound choice for quantitative reference materials.

Here, we report the development, characterization, and multi-laboratory certification of the first bovine-derived single-copy genomic DNA CRM, targeting the nuclear *β-actin* gene. We selected *β-actin* as the target because it is a well-conserved, experimentally validated single-copy nuclear gene with established primer–probe systems [21], ensuring immediate compatibility with existing laboratory workflows. We comprehensively evaluated the CRM’s homogeneity and short-term, long-term, and freeze–thaw stability in accordance with ISO 33405:2024 [22] and JJF 1343-2022 [23]. The certified copy number concentration was assigned through a collaborative study involving nine independent laboratories, each employing the same dPCR protocol. This work provides a systematic framework to enhance data comparability and measurement accuracy in meat adulteration testing and establishes a template for developing single-copy DNA CRMs for other animal species.

## 2. Results

### 2.1. Quality Assessment and Evaluation of Genomic DNA

**Figure 1:**
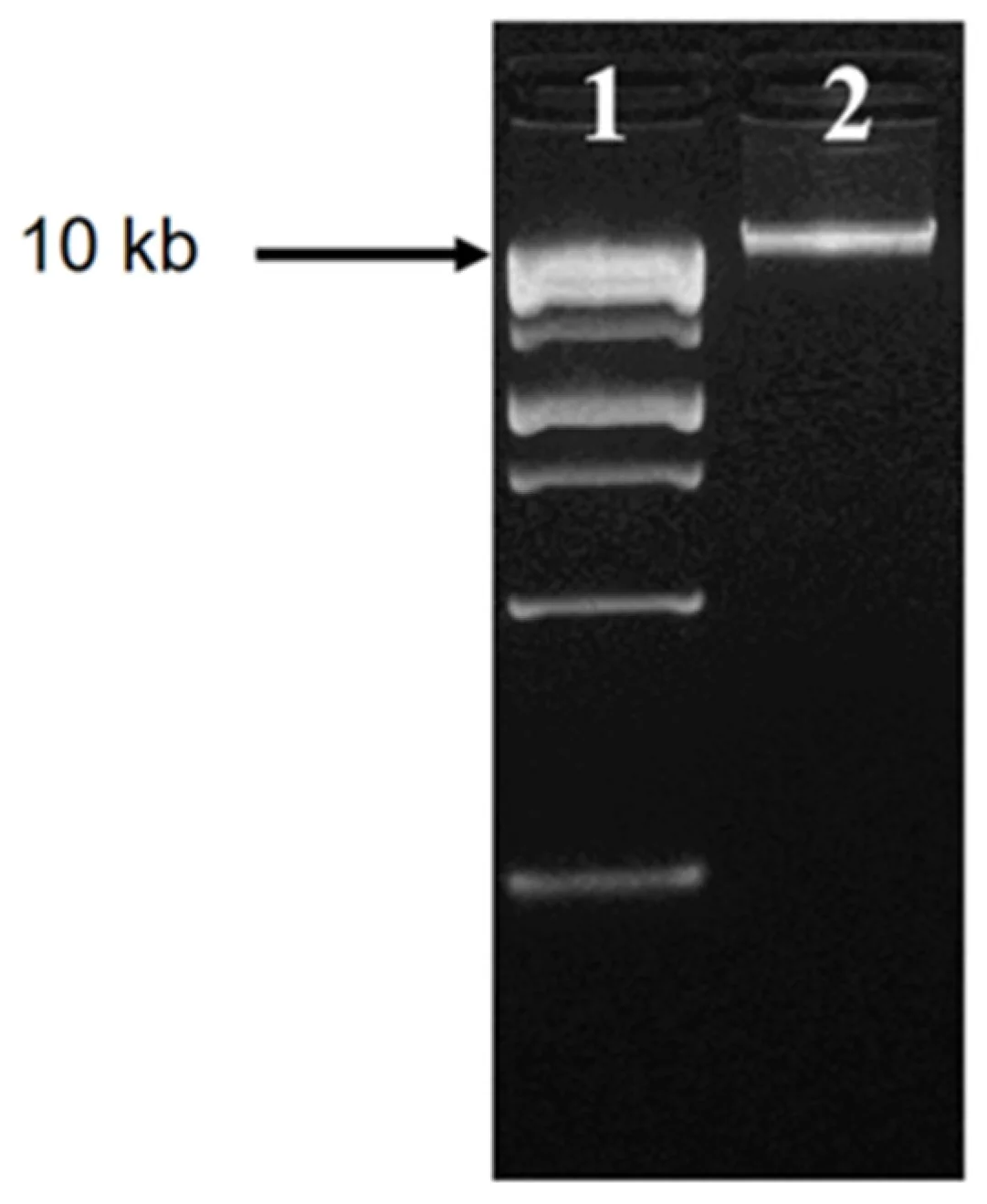
Electrophoresis of bovine genomic DNA. Lane 1: 10 Kb marker; Lane 2: bovine genomic DNA.

The purity of the extracted bovine genomic DNA was evaluated using 1% agarose gel electrophoresis. Following electrophoresis at 120 V for 20 min, the resulting gel was imaged using a gel documentation system (Figure 1). The electrophoresis revealed a single, distinct band of genomic DNA without any smearing, indicating that the DNA quality was suitable for the experimental requirements. The quality and concentration of the genomic DNA were further assessed using a Nanodrop™ 2000 spectrophotometer and a Qubit 2.0 fluorometer, respectively. The OD260/OD280 ratio was 1.98, the A260/A230 ratio was 2.06, and the concentration was determined to be 206 ng/μL. These results collectively confirm that the extracted genomic DNA meets the necessary preparation standards for subsequent analyses. 

### 2.2. Homogeneity Assessment

A total of 200 units of each CRM (high values and low values) were prepared; to assess homogeneity, 12 units were randomly selected, and three replicates were taken from each one. Absolute quantification of the samples was performed using the ddPCR method. The calculated F-values did not exceed the critical value of 2.18 at the 95% confidence level, confirming the homogeneity of the samples (Table 1).

### 2.3. Stability Assessment

**Figure 2:**
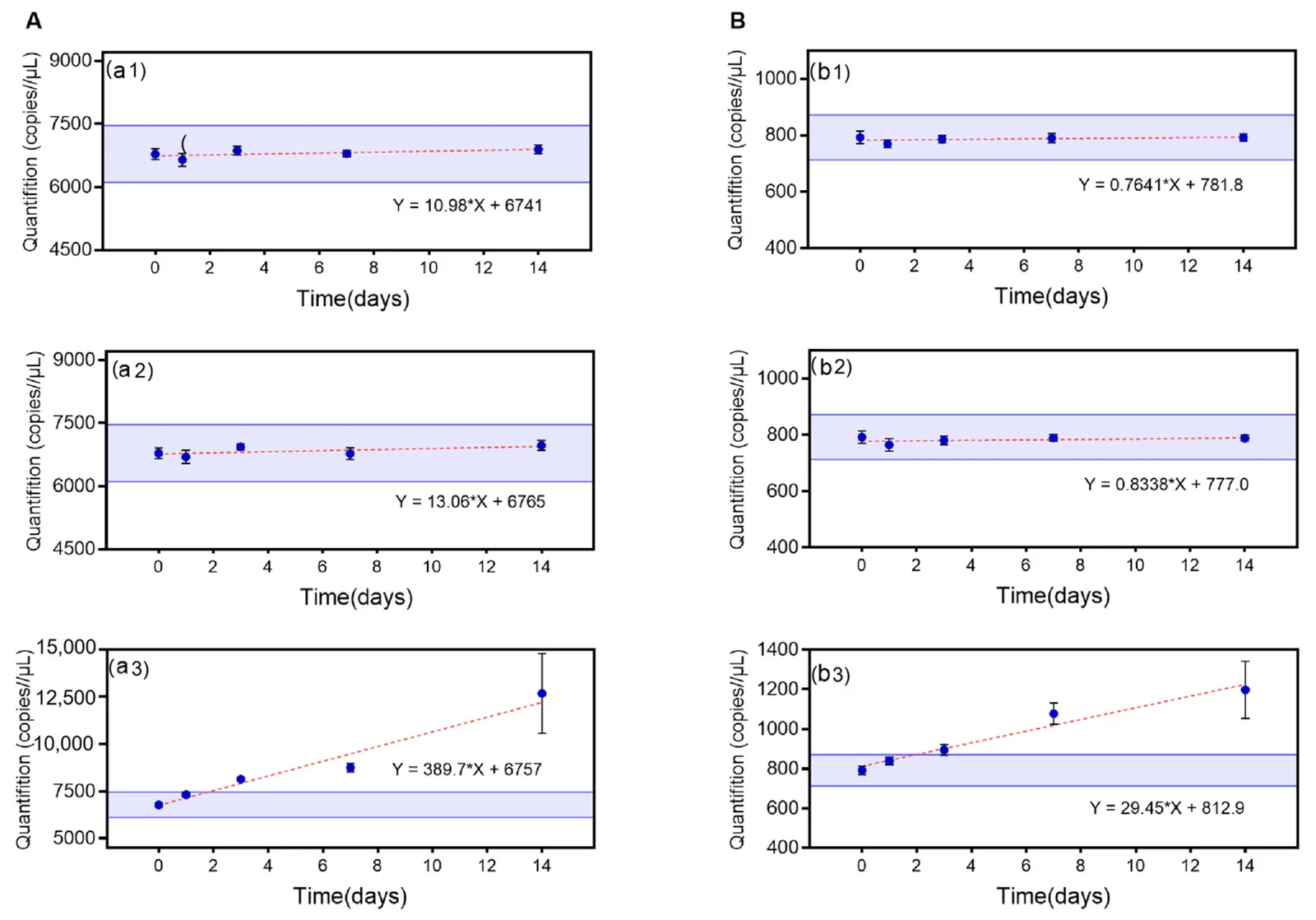
Trends and concentrations of short-term stability for candidate RMs: (**A**) Short-term stability of high-value candidate RMs at 4 °C (**a1**), 25 °C (**a2**), and 60 °C (**a3**). (**B**) Short-term stability of low-value candidate RMs at 4 °C (**b1**), 25 °C (**b2**), and 60 °C (**b3**). The purple area represents the expanded uncertainty interval for the certified value of the copy number concentration.

Short-term stability (transport stability) is a property of the CRMs that describes their stability under expected transport conditions. High temperatures cannot be ruled out during transportation. Therefore, CRM samples were stored at 4 °C, 25 °C, and 60 °C for 0, 1, 3, 7, and 14 days. Three tubes were randomly selected at each temperature, and each tube was tested in three replicates by ddPCR. The results of the *t*-test showed no significant differences in the slope (β1) between the CRMs stored for two weeks at 4 °C or 25 °C at a 95% confidence level. However, at 60 °C, the *β-actin* gene number significantly increased during storage (Figure 2). These results of short-term CRM stability analysis indicated that bovine-derived genomic DNA is stable under room-temperature (4 °C, 25 °C) storage and transport for up to 14 days, though we advise cold chain transportation to minimize the likelihood of differences in the measured values.

**Figure 3:**
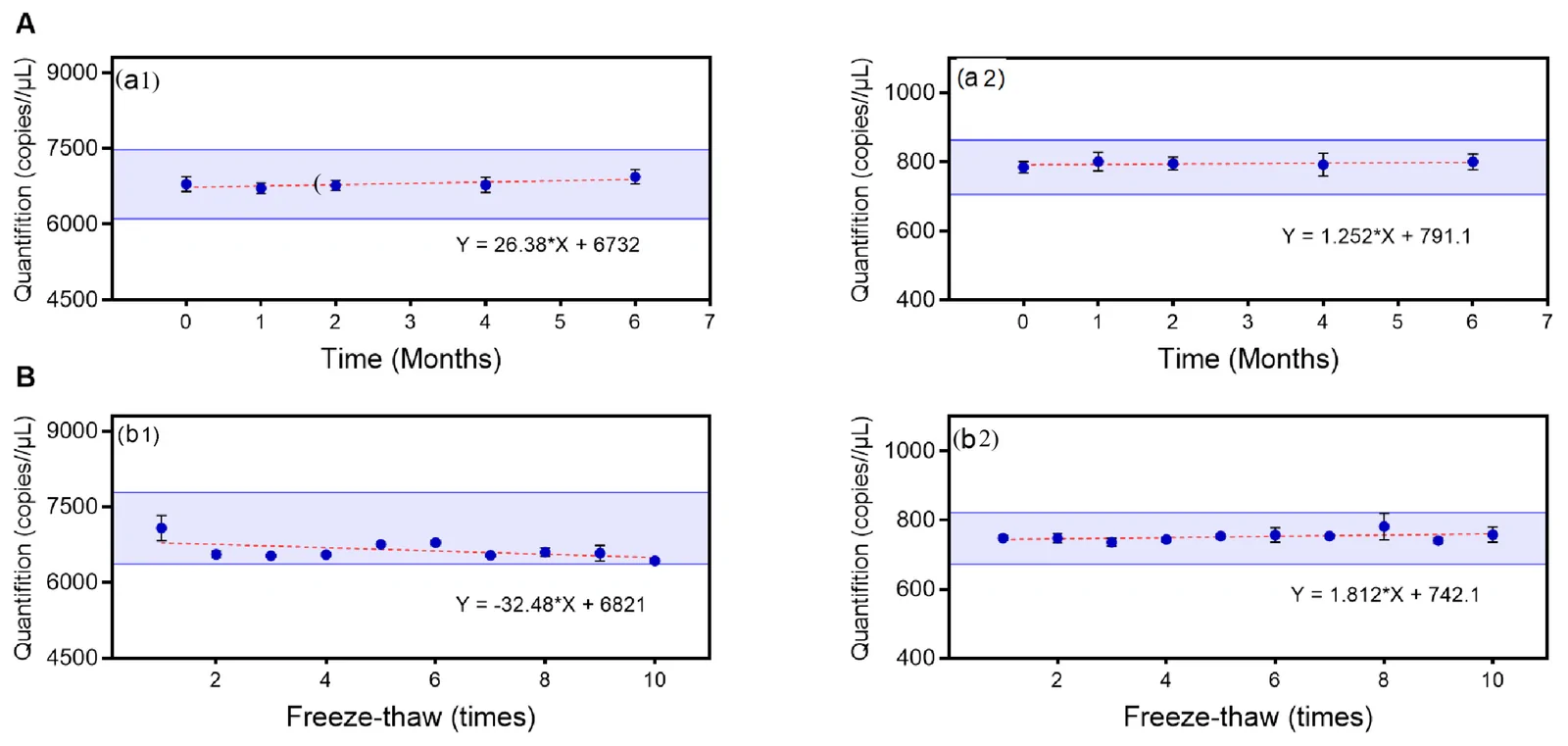
Trends and concentrations of long-term stability and freeze–thaw stability for candidate RMs: (**A**) Trends and concentrations for long-term stability analysis of high-value (**a1**) and low-value (**a2**) candidate RMs. (**B**) Trends and concentrations for freeze–thaw stability (10 cycles) analysis of high-value (**b1**) and low-value (**b2**) candidate RMs. The purple area represents the expanded uncertainty interval for the certified value of the copy number concentration.

To determine the long-term stability of the CRM, three tubes of CRM samples were randomly selected and stored at -20 °C. Absolute quantitative analysis of the *β-actin* gene was performed at 0, 1, 2, 4, and 6 months using ddPCR technology. The analysis results (Figure 3A) indicate that this batch of CRMs can be stably stored at −20 °C for 6 months without significant differences in measured values.

Finally, the freeze–thaw cycles on the *β-actin* copy number were assessed. The CRMs were subjected to 10 freeze–thaw cycles, and the data were analyzed using a *t*-test. The results (Figure 3B) indicate that after 10 freeze–thaw cycles, the measured values of the CRM showed no significant upward or downward trend. Therefore, it can be concluded that the bovine *β-actin* gene DNA CRM maintains stable measured values after 10 freeze–thaw cycles.

### 2.4. Collaborative Characterization Results

To ensure the reliability of the results, 9 laboratories with extensive experience in nucleic acid testing employed dPCR technology to determine the bovine-derived *β-actin* samples. Each laboratory received two tubes of each concentration of CRM samples (high values and low values), with each tube undergoing four replicate tests. Consequently, each laboratory provided 16 data points, and copy number evaluation and analysis were conducted according to ISO 33405:2024 standards. Statistical analysis indicated (Table 2) that the data followed a normal distribution, with all values falling within the 95% confidence level. Furthermore, no significant differences were observed between the mean values of the laboratories. Based on this, the validated arithmetic mean copy number of the *β-actin* gene in bovine-derived CRMs was estimated to be 6.85 × 10^3^ copies/μL (high values) and 8.00 × 10^2^ copies/μL (low values).

### 2.5. Uncertainty Evaluation

The uncertainty of the CRMs for the *β-actin* gene was estimated in accordance with ISO 33405:2024. The uncertainty of the assigned values is influenced by three parameters: quantification method, homogeneity, and stability. The expanded uncertainty of the assigned value is 0.63 × 10^3^ copies/μL for high-value CRM and 0.77 × 10^2^ copies/μL for low-value CRM, as presented in Table 3.

### 2.6. Suitability Testing of CRMs

#### 2.6.1. Suitability of Bovine-Derived *β-Actin* Gene DNA CRMs for Standards

**Figure 4:**
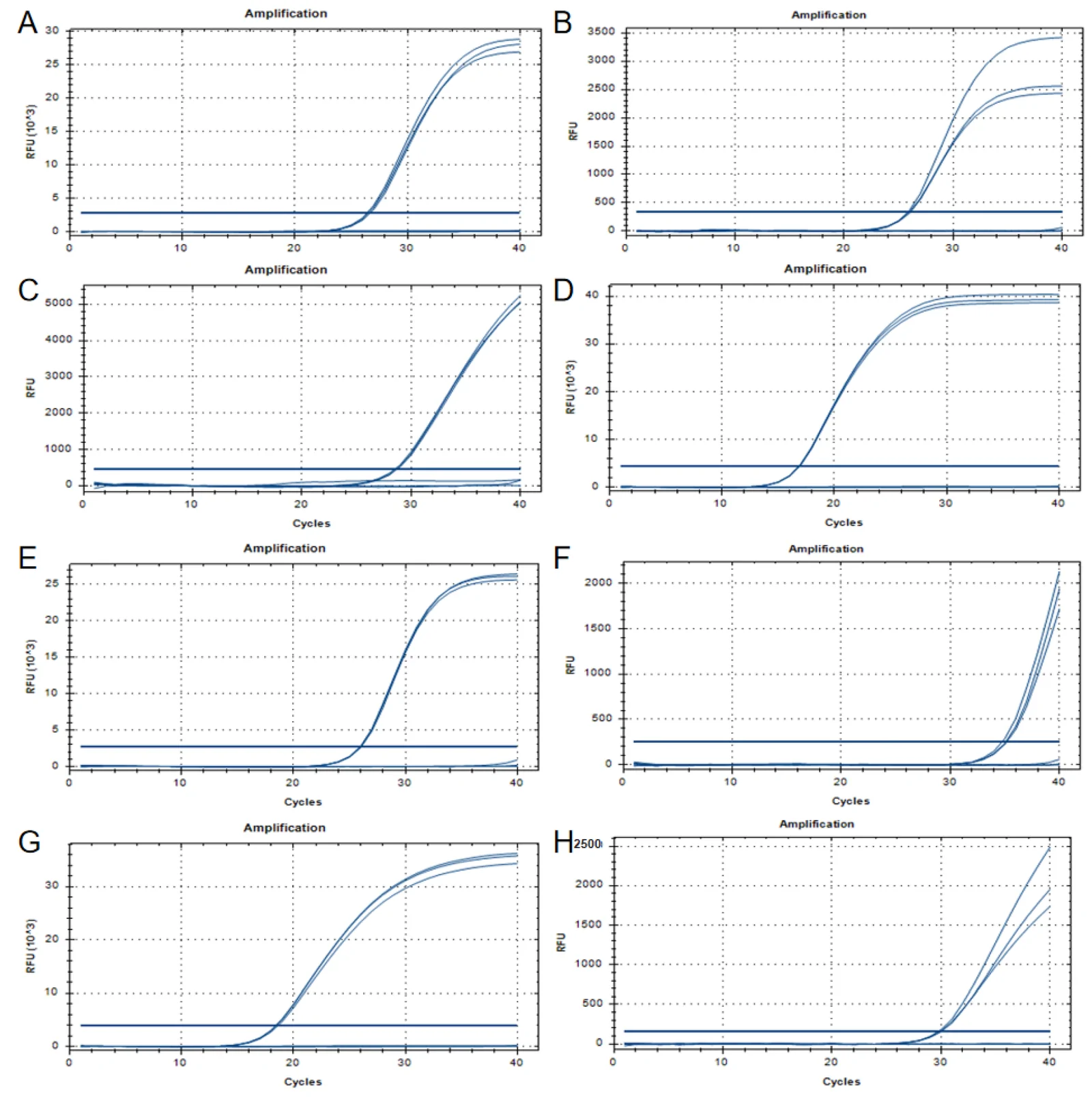
Validation of the standard suitability of bovine-derived *β-actin* gene DNA CRMs using qPCR: (**A**) SN/T 2557-2010 standard. (**B**) SN/T 2557-2010 standard. (**C**) SZDB/Z 268-2017 standard. (**D**) NY/T 1946-2010 standard. (**E**) GB/T 25165-2010 standard. (**F**) GB/T 25165-2010 standard. (**G**) GB/T 38164-2019 standard. (**H**) ISO/TS 20224-1:2020 standard.

To validate the suitability of the CRMs, we systematically collected and organized the existing primer and probe sequences suitable for bovine-derived component detection (Table 5). Results (Figure 4) demonstrate that these CRMs achieve effective amplification in qPCR, indicating their suitability for relevant standards.

#### 2.6.2. Analysis of Adulteration Models and Quantitative Bias in Single- and Multi-Copy Genes

**Figure 5:**
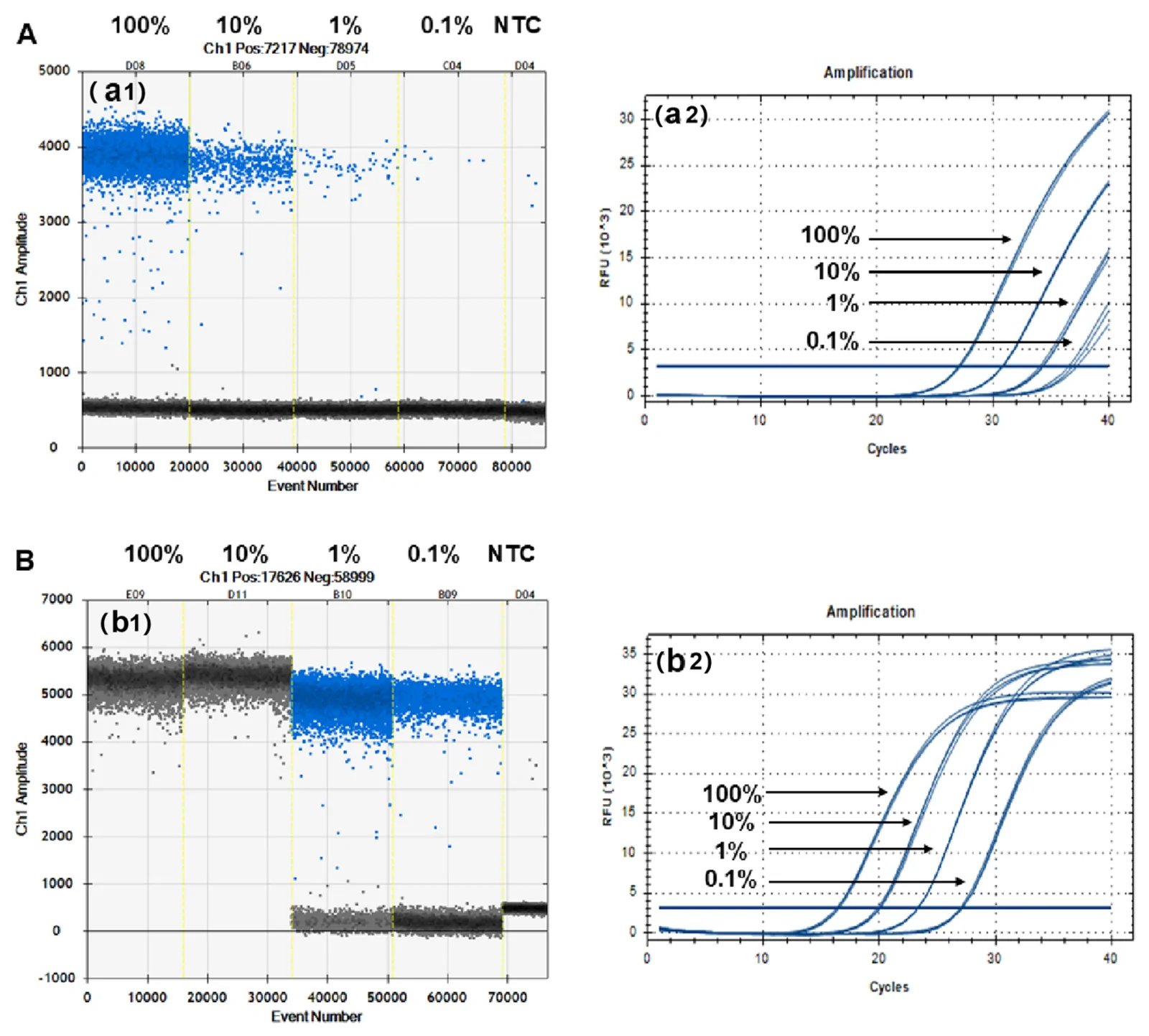
Quantitative deviation test of single-copy *β-actin* gene and multi-copy *cytb* gene in bovine-derived components: (**A**) Detection results of single-copy *β-actin* gene at different adulteration ratios using dPCR (**a1**) and qPCR (**a2**). (**B**) Detection results of the multi-copy *cytb* gene at different adulteration ratios using dPCR (**b1**) and qPCR (**b2**).

Bovine-derived DNA was spiked into duck-derived DNA, and quantitative analysis of the *β-actin* gene (single-copy gene) and *cytb* gene (multiple-copy gene) was performed using dPCR and qPCR. Results showed that the single-copy *β-actin* gene exhibited a good linear relationship across different dilution ratios (Figure 5A(a1)). When the dilution ratio is 0.1%, a qPCR CT value of 37 is considered negative (Figure 5A(a2)).

For the multi-copy *cytb* gene, positive and negative droplets were successfully separated, and signal output was achieved at 0.1% and 1% dilution ratios. However, at 10% and 100% dilution ratios, excessive copy numbers exceeded the upper LOD, preventing signal output (Figure 5B(b1)). In qPCR, the amplification curve for the multi-copy *cytb* gene exhibited a distinct leftward shift compared to that of the single-copy *β-actin* gene (Figure 5B(b2)). The quantitative deviation between single- and multi-copy genes was approximately 500-fold (Table 4).

#### 2.6.3. Real Sample Test

**Figure 6:**
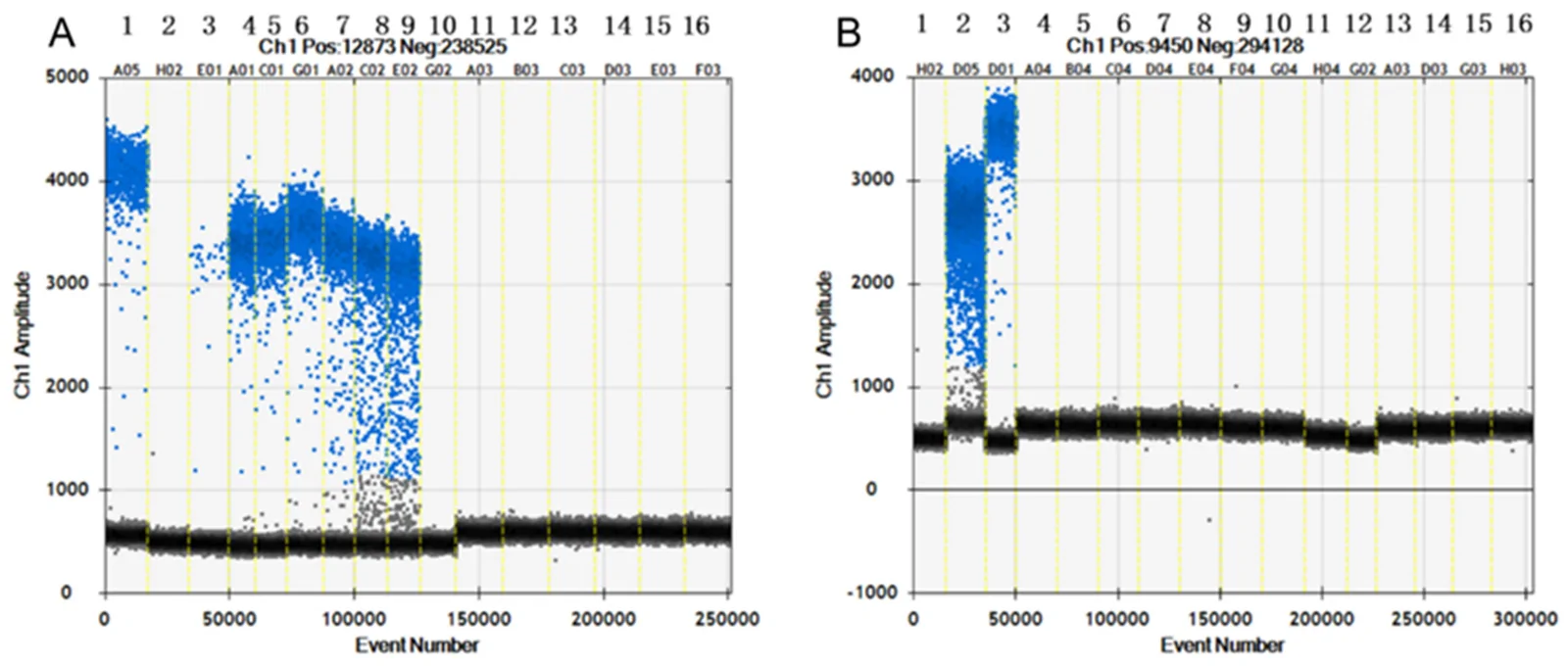
Bovine-derived CRMs were used as positive control materials to monitor bovine-derived components in commercial samples: (**A**) The pair of primers and probes for bovine-derived CRMs was tested on 16 samples. (**B**) The pair of primers and probes for duck-derived CRMs was tested on 16 samples. From left to right, bovine-derived CRMs, duck-derived CRMs, beef skewers, beef rolls 1 and 2, beef meatballs 1 and 2, dried beef 1 and 2, lamb rolls 1 and 2, lamb meatballs 1 and 2, lamb skewers 1 and 2, and ddH_2_O.

Using bovine-derived CRMs as positive control samples, 13 commercially available samples were tested for bovine components. Results showed that the ingredient information on 12 samples matched their labels. Among these, Sample 3 exhibited a low number of bovine-derived positive droplets (Figure 6A) but a high number of duck-derived positive droplets (Figure 6B), indicating significant adulteration of this beef skewer sample with duck meat and constituting food fraud.

## 3. Discussion

The development of metrologically sound reference materials is a prerequisite for standardizing quantitative measurements in food authenticity testing. In this study, we developed, rigorously characterized, and certified the first bovine-derived single-copy genomic DNA CRM targeting the nuclear *β-actin* gene (GBW(E)091323). Unlike existing bovine DNA standards, which are predominantly qualitative or semi-quantitative calibrants based on multi-copy gene targets, the CRM reported here provides a traceable copy-number concentration linked to genome equivalents through a well-characterized single-copy nuclear locus.

The central analytical finding of this work—the approximately 500-fold quantitative deviation between single-copy (*β-actin*) and multi-copy (*cytb*) gene targets—has significant implications for the meat testing community. Most current regulatory detection standards for bovine-derived ingredients, including SN/T 2557-2010, GB/T 25165-2010, and GB/T 38164-2019, specify multi-copy mitochondrial or ribosomal DNA targets to maximize sensitivity. While this strategy is effective for qualitative detection, our data demonstrate that it introduces profound quantitative inaccuracy. The *cytb* gene, located in the mitochondrial genome, is present at variable copy numbers that depend on tissue type, cell type, metabolic state, and even the age of the animal. Consequently, a *cytb*-based copy number measurement cannot be reliably converted to a mass fraction or percentage of adulteration without a tissue-specific, empirically determined conversion factor. This is not a subtle effect: the 185- to 569-fold deviation we observed renders multi-copy gene data effectively non-quantitative when the goal is to determine the proportion of an adulterating species.

These findings align with and extend earlier observations in other species. Multi-copy target bias has been documented in nucleic acid quantification, and the variable copy number of mitochondrial genes across bovine tissues has been noted in previous studies [24,25]. However, to our knowledge, this is the first study to systematically quantify the resulting measurement bias in a realistic adulteration model using a certified single-copy reference material as the benchmark. The CRM we developed thus serves a dual purpose: as a calibration standard for single-copy quantification and as a diagnostic tool to assess the quantitative accuracy of existing and future detection methods.

The stability profile of the CRM warrants careful interpretation. Our data demonstrate robust stability under recommended storage (−20 °C, ≥6 months) and transport (4–25 °C, ≥14 days) conditions. The observed increase in apparent copy number at 60 °C is noteworthy. Elevated temperature promotes DNA denaturation and partial fragmentation, which in dPCR can alter the Poisson statistics underlying copy-number estimation: fragmented DNA may partition across multiple droplets, leading to overestimation. This mechanism has been described for other DNA reference materials subjected to thermal stress [26], and it underscores the importance of cold-chain transport for CRMs intended for quantitative applications. The practical recommendation emerging from these data is that while the CRM tolerates brief excursions to ambient temperature without significant degradation, sustained exposure to extreme heat must be avoided.

The freeze–thaw stability (≥10 cycles) is a practically important result, as it indicates that end-users can aliquot the CRM upon receipt and thaw individual aliquots for single-use without concern for cumulative degradation. This finding reduces the risk of measurement error arising from repeated freeze–thaw cycling during routine laboratory use. The collaborative characterization study, encompassing nine independent laboratories, provides strong evidence for the inter-laboratory reproducibility of the dPCR-based measurement protocol. A relative standard deviation of 3.7–4.2% across laboratories is consistent with or better than values reported for other DNA CRMs characterized by dPCR [27,28]. The dominant source of uncertainty was the characterization step itself (u_char,rel_ ≈ 3.2–3.4%), suggesting that future reductions in overall uncertainty may be achieved through further refinement of the dPCR protocol—for example, by adopting higher-precision droplet volume calibration or automated droplet generation systems.

Several limitations of this study should be acknowledged. First, the CRM was prepared from a single tissue source (hindquarter muscle) of a single breed (Angus); the generalizability of the certified *β-actin* copy number to other bovine tissues and breeds, while expected based on the single-copy nature of the target, was not experimentally verified across a broad panel. Second, the real-sample testing was limited to 13 commercial products from a single geographic market; a larger, multi-regional sampling would strengthen the generalizability of the CRM’s practical utility. Third, the long-term stability study extends only to 6 months; ongoing monitoring has been required to establish the full shelf life of the CRM under recommended storage conditions. Fourth, while the CRM was shown to be compatible with eight bovine detection standards, the quantitative accuracy of those standards when used with the CRM was not exhaustively characterized beyond the *β-actin*/*cytb* comparison.

The implications of this work extend beyond the specific CRM reported here. The demonstration that multi-copy gene targets can introduce systematic quantitative bias of up to ~500-fold argues for a re-evaluation of current detection standards. We suggest that the meat testing community should move toward single-copy nuclear gene targets for quantitative applications, reserving multi-copy targets for qualitative screening where maximum sensitivity is required. The framework established in this study—combining a single-copy CRM, a standardized dPCR protocol, multi-laboratory validation, and real-world sample verification—can serve as a template for developing analogous CRMs for other high-risk species in the food supply chain, including ovine, porcine, and avian sources.

## 4. Materials and Methods

### 4.1. Extraction and Evaluation of Genomic DNA

The beef samples used in this study were derived from the hindquarters of Angus cattle (Aberdeen Angus), which were procured from Inner Mongolia University. Genomic DNA was extracted from these samples using the Animal Tissue DNA Extraction Kit provided by Hangzhou Simgen Biotechnology Co., Ltd. (Hangzhou, China). The integrity of the extracted DNA was subsequently verified via 1% agarose gel electrophoresis. The concentration and purity of the genomic DNA were evaluated using a Nanodrop™ 2000 spectrophotometer (Thermo Fisher, Waltham, MA, USA), and the quantity was further confirmed with a Qubit 2.0 Fluorometer (Thermo Fisher, Waltham, MA, USA).

### 4.2. Digital PCR Assay

**Figure 7:**
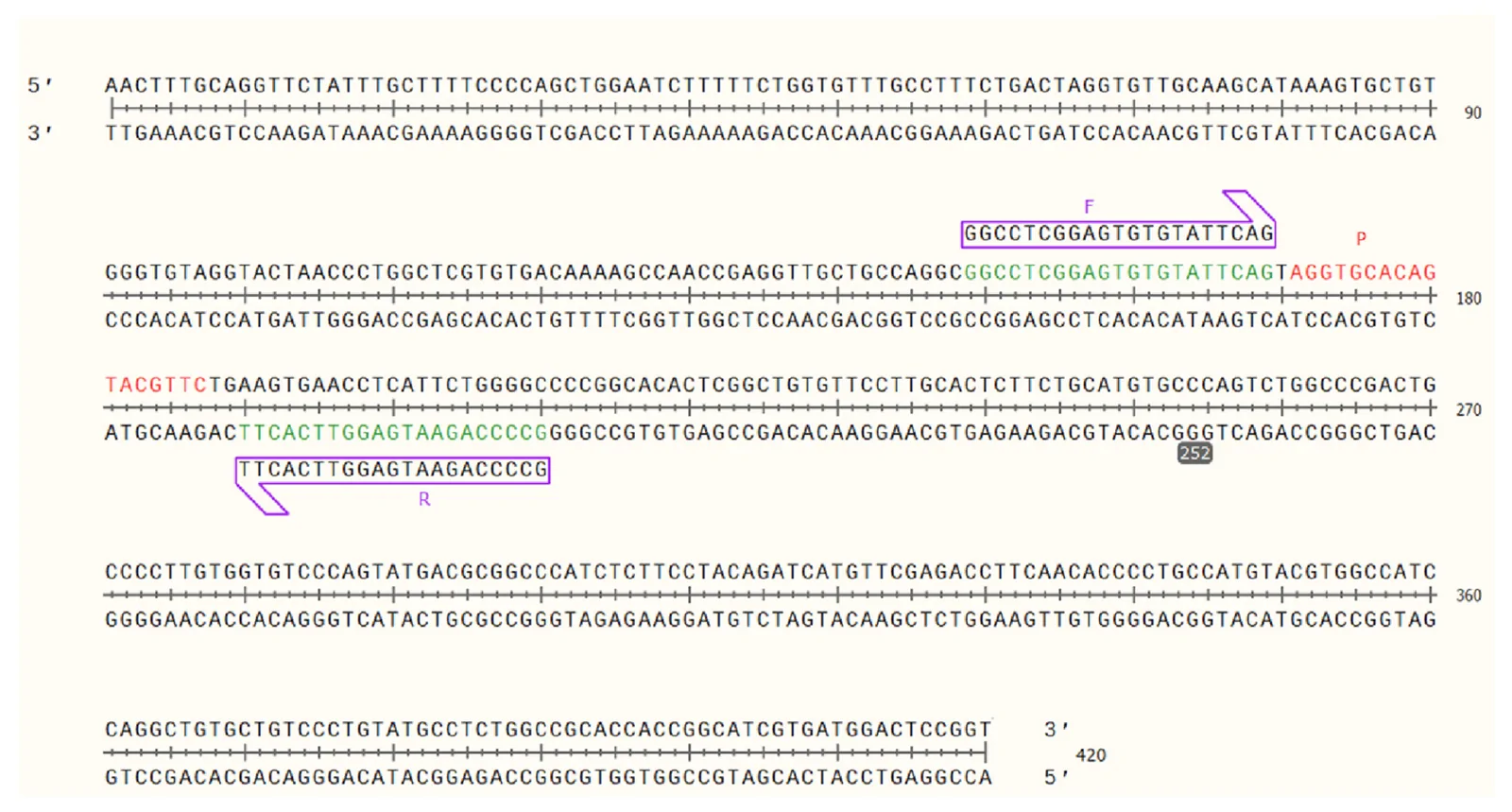
Sequence of the beta-actin gene (NC_037352.1). The primers (F,R) and probe (P) are shown. The amplification size was 62 bp.

The target gene is a single-copy nuclear gene (*β-actin*) in the genome. The primers and probe (Figure 7) used for specific quantification were as described previously [21] and synthesized by Sangon Biotech (Shanghai, China) Co., Ltd. (Shanghai, China). The primer and probe sequences are shown in Table 5.

In this study, droplets were generated using the Bio-Rad QX200 droplet digital PCR system (Bio-Rad Laboratories, Inc., Hercules, CA, USA), which combines oil-in-water emulsification technology with microfluidics. The core principle is as follows: under vacuum-driven aspiration, the sample (aqueous phase) and the oil phase are mixed within microfluidic channels. Surface tension and shear forces partition the sample into a large number of uniform, nanoliter-sized water-in-oil droplets. Approximately 20,000 droplets are generated per sample, each with a volume of about 1 nL, allowing independent PCR reactions within each droplet. The droplet generation process is rapid, with 8 samples processed within 3 min. The generated droplets are then transferred to a 96-well PCR plate and amplified in a conventional thermal cycler.

In this study, the critical parameters that influence dPCR performance were thoroughly optimized, including annealing temperature, cycle number, and primer/probe concentrations. The optimized dPCR reaction mixture consisted of 10 µL of 2× ddPCR Supermix for Probes (Bio-Rad Laboratories, Inc., Hercules, CA, USA), 1 µL of each primer (10 µM), 0.5 µL of probe (10 µM), 2 µL of DNA template, and 5.5 µL of ddH_2_O. The thermal cycling conditions were 95 °C for 10 min, followed by 40 cycles consisting of 94 °C for 15 s and 60 °C for 1 min, then 98 °C for 10 min and held at 4 °C, with a temperature increase and decrease rate of 2 °C/s. Fluorescent signals from the amplified droplets were individually captured in the QX200 droplet reader (Bio-Rad, US) and analyzed using QuantaSoft Version 1.7.4 software.

### 4.3. Assessment of Homogeneity

A total of 200 units of each CRM (high values and low values) were prepared, in which the high and low values refer to copy numbers corresponding to sample concentrations of 20.06 ng/μL and 2.36 ng/μL, respectively. To verify the consistency of measurement values for CRMs across different bottles, 12 units were randomly selected in accordance with the technical requirements of ISO 33405:2024. Three subsamples were taken from each unit and measured using dPCR. F-test analysis was employed to determine the measurement values of the bovine genomic *β-actin* gene, followed by a statistical significance comparison.

### 4.4. Assessment of Stability Monitoring

Stability is a critical parameter for evaluating CRMs. Stability assessments include short-term stability (transport stability), long-term stability, and freeze–thaw stability. The statistical models are based on the linear regression and random-effects models specified in JJF 1343-2022 [23], Valuation and Assessment of Homogeneity and Stability of Reference Materials, and ISO 33405:2024 [22].

For short-term stability assessment, CRMs were stored in constant-temperature chambers maintained at 4 °C, 25 °C, and 60 °C. Stability monitoring was conducted on days 1, 3, 7, and 14 at each temperature and time point; three samples were taken, for a total of 36 samples, with three replicates performed for each sample. A synchronous stability monitoring approach was used, which ensured the consistency of samples from Day 0 (short-term stability), Month 0 (long-term stability), and the first freeze–thaw cycle, thereby establishing a common baseline for comparisons across all time points. These samples were tested alongside samples stored long-term at −20 °C (Day 0).

To evaluate the long-term stability of the CRMs, random sampling was performed at 0, 1, 2, 4, and 6 months for those stored at −20 °C. Each sampling event involved the retrieval of three vials, with three replicates per vial.

To ensure the stability of these CRMs, which were the DNA solutions used in the study, the freeze–thaw stability needs to be evaluated. One DNA solution of the CRMs stored at −20 °C was randomly selected, thawed at room temperature until completely dissolved, and then refrozen at −20 °C for at least 12 h. This procedure was repeated 10 times. Statistical analysis using a *t*-test was conducted to evaluate the short-term, long-term, and freeze–thaw stability of the CRMs.

### 4.5. Collaborative Characterization

To evaluate the variability of the reference material across different laboratories for the purpose of assigning property values. According to the requirements of JJF1343-2022, for the joint value assignment of certified reference materials, the number of participating laboratories is usually no fewer than eight. The property values of this batch of CRMs were determined by 9 laboratories, each utilizing dPCR for the measurements. These laboratories have extensive experience in DNA measurement and are highly regarded in their field. In the interlaboratory study, each of the nine participating laboratories received two vials at each concentration level (high and low) and determined the copy number concentration using the specified dPCR protocol, with each vial analyzed in quadruplicate. The 9 participants needed to export the data and original files to the organization’s laboratory. The returned data were analyzed in accordance with ISO 33405:2024 requirements.

### 4.6. Assigned Value and Uncertainty Assessment

The Dixon and Grubbs test was used to identify and remove any outliers. The dPCR method was used for the quantitative analysis, with the arithmetic mean of the measurement results serving as the designated value.

The uncertainty of bovine-derived *β-actin* genomic DNA CRMs involved several sources: value assignment-induced uncertainty ($u_{\mathrm{char},\mathrm{rel}}$), homogeneity-induced uncertainty $u_{\mathrm{bb},\mathrm{rel}}$, and stability-induced uncertainty ($u_{\mathrm{lst},\mathrm{rel}}$, $u_{\mathrm{sts},\mathrm{rel}}$) The combined standard uncertainty ($u_{\beta -\mathrm{actin},\mathrm{rel}}$) was calculated using Formula (1). The expanded uncertainty ($u_{\mathrm{CRM},\mathrm{rel}}$) of the CRM was determined at a 95% confidence level, with an expansion factor k equal to 2, in accordance with Formula (2):

(1) $$u_{\beta -\mathrm{actin}}\;=\;\sqrt{u_{\mathrm{char},\mathrm{rel}}^{2}\;+{\;u}_{\mathrm{bb},\mathrm{rel}}^{2}\;+\;u_{\mathrm{lts},\mathrm{rel}}^{2}\;+\;u_{\mathrm{sts},\mathrm{rel}}^{2}}$$

(2) $$U_{\mathrm{CRM},\mathrm{rel}}=k\;\times {\;u}_{c}\;(k=2,\;95\%\;confidence\;level)$$

### 4.7. Suitability Testing of CRMs

#### 4.7.1. Suitability of Bovine Genomic DNA CRMs for Standards

Primer–probe pairs were selected from relevant standards for bovine-derived component detection (Table 5). The suitability of the bovine CRMs was subsequently verified through qPCR amplification performed in accordance with each standard’s protocol.

**Table 5:** Bovine-derived primer–probe pairs in the standard.

Type	Sequence (5′ to 3′)	Gene	Standards
1	CCTCAGCAGGGTCTTCACCA	*GH-BGH*	SN/T 2557-2010 [18]
	CGTGTCAGGATTGGGTAAT		
	FAM-CAGCCTGGTGTTTGGCACCTCGGA-TAMRA		
2	CCGATGGATGTGTTCAGAGCT	*GH-BGH*	SN/T 2557-2010 [18]
	GCCAAATGTCTGGGTGTAGATACC		
	FAM-TGGGCTTTAGGGCTTCCGAATGTGAA-TAMRA		
3	C(AT)TACACCTTCCTAGAAACATGAAA(CT)	*Cytb*	SZDB/Z 268-2017 [29]
	TGG(CT)AGTAC(AG)TA(CT)CCTAT(AG)AATGCTGT(AG)		
	FAM-TAATCCTTCTGCTCAC-BHQ1		
4	GGGCTGATTAGTCATTAGTCCAT	*D-Loop*	NY/T 1946-2010 [30]
	CAGACATCTGGTTCTTTCTTCAGGGC		
	FAM-TGGACCGTTTTAGAGAGA-MGB		
5	CCGATGGATGTGTTCAGAGCT	*GH*	GB/T 25165—2010 [19]
	GCCAAATGTCTGGGTGTAGATACC		
	FAM-TGGGCTTTAGGGCTTCCGAATGTGAA-TAMRA		
6	CCTGAGAAACGGCTACCAT	*18S* rRNA	GB/T 25165—2010 [19]
	CGTGTCAGGATTGGGTAAT		
	FAM-TGCGCGCCTGCTGCCTTCCT-TAMRA		
7	TGGAGTAATCCTTCTGCTCACAGT	*Cytb*	GB/T 38164-2019 [20]
	TGACTGTTGCTCCTCAGAATGATA		
	FAM-AGCATTTATAGGATACGTCC-MGB NFG		
8	GGCCTCGGAGTGTGTATTCAG	*β-actin*	ISO/TS 20224-1:2020 [18]
	GCCCCAGAATGAGGTTCACTT		
	FAM-AGGTGCACAGTACGTTC-NFQ-MGB		

#### 4.7.2. Analysis of Quantitative Deviation in the *β-Actin* Gene and *Cytb* Gene in Bovine-Derived Components

Given that most bovine-derived component detection methods target multi-copy genes, which may introduce quantitative bias. Fresh bovine-derived genomic DNA was spiked into the duck-derived DNA background at volume ratios of 0.1%, 1%, 10%, and 100%, with both DNAs adjusted to the same concentration. dPCR quantification was performed using corresponding primers targeting the bovine *β-actin* and *cytb* genes (Table 5 and Table 6) to compare quantitative deviations between single- and multi-copy genes.

#### 4.7.3. Real Sample Test

To validate the effectiveness of the detection system, this study employed bovine-derived genomic DNA CRMs as positive controls for testing commercially available beef products. To safeguard assay specificity and account for common substitution practices, duck-derived DNA CRMs were incorporated as negative controls alongside the bovine CRMs. Using specific primers and probes designed for bovine and duck-derived DNA standards, dPCR amplification was performed on both the bovine and duck-derived DNA standards, as well as on 13 commercially available samples (including beef and lamb rolls, meat skewers, jerky, and meatballs). The primers and probes are shown in Table 7. The testing system and procedures are consistent with those in Section 4.2.

## 5. Conclusions

In this study, we successfully developed bovine *β-actin* gene DNA RM (GBW(E)091323), which has been confirmed by China’s State Administration for Market Regulation. Nine qualified laboratories were invited to conduct a comprehensive validation and certification of this RM using dPCR platforms. Validation results confirmed that the high-value CRM has a copy number of (6.85 ± 0.63) × 10^3^ copies/μL, while the low-value CRM has a copy number of (8.00 ± 0.77) × 10^2^ copies/μL. Regarding stability assessment, the CRM remained stable for 14 days under cold chain transportation conditions. During the end-user application, the value showed no significant change after 10 freeze–thaw cycles. It also maintained stability for 6 months at −20 °C. Furthermore, the CRM passed the standard applicability evaluation and is suitable for current Chinese standards. Analysis of multi-copy quantitative deviation revealed a deviation of approximately 500-fold between the two. The development of this bovine *β-actin* gene DNA CRM fills a gap in bovine single-copy gene DNA standards. It provides a crucial tool for validating detection methods and conducting proficiency testing for bovine content in meat and meat products, holding significant scientific and practical value.

## Figures and Tables

**Table 1:** Results of the homogeneity analysis: high and low values.

*β-actin*	$v_{1}$	$v_{2}$	$M_{\mathrm{between}}$	$M_{\mathrm{within}}$	F	*F*_0.05(11,24)_	${u_{\mathrm{bb}}=s}_{\mathrm{bb}}$	$u_{\mathrm{bb},\mathrm{rel}}$
High Values	11	24	2.63 × 10^4^	1.52 × 10^4^	1.73	2.18	60.8	0.91%
Low Values	11	24	1.28 × 10^3^	7.89 × 10^2^	1.62	2.18	12.8	1.7%

**Table 2:** Results for dPCR quantification of the *β-actin* gene copy number by 9 independent participating laboratories.

Laboratories	Bovine-Derived *β-actin* Gene CRMs	
	High Values (Copies/μL)	Low Values (Copies/μL)
1	7.13 × 10^3^	8.52 × 10^2^
2	6.99 × 10^3^	7.92 × 10^2^
3	6.64 × 10^3^	8.02 × 10^2^
4	6.40 × 10^3^	7.62 × 10^2^
5	6.94 × 10^3^	7.72 × 10^2^
6	7.21 × 10^3^	7.69 × 10^2^
7	6.81 × 10^3^	7.79 × 10^2^
8	6.66 × 10^3^	8.45 × 10^2^
9	6.86 × 10^3^	8.28 × 10^2^
Mean	6.85 × 10^3^	8.00 × 10^2^
SD	2.55 × 10^2^	3.38 × 10^1^
RSD%	3.7	4.2

**Table 3:** Analysis of uncertainty for bovine-derived CRMs.

CRMs	$u_{\mathrm{char},\mathrm{rel}}$	$u_{\mathrm{bb},\mathrm{rel}}$	$u_{\mathrm{sts},\mathrm{rel}}$	$u_{\mathrm{lts},\mathrm{rel}}$	$u_{\beta -\mathrm{actin},\mathrm{rel}}$	$u_{\mathrm{CRM},\mathrm{rel}}$	*U_CRM_* (k = 2)/ Copies/μL
High Values	3.2%	0.9%	1.9%	2.3%	4.6%	9.2%	(6.85 ± 0.63) × 10^3^
Low Values	3.4%	1.7%	2.0%	2.1%	4.8%	9.6%	(8.00 ± 0.77) × 10^2^

**Table 4:** Comparison of detection limits and quantification deviations for single-copy (*β-actin*) and multi-copy (*cytb*) genes in a model for adulteration with bovine-derived ingredients.

Adulteration Ratio (*v*/*v*)	*β-actin* (Copies/μL)	*cytb* (Copies/μL)	Deviation Factor (*cytb*/*β-actin*)
0.10%	11	2093	185
1%	42	23,907	569
10%	378	No Call	/
100%	4673	No Call	/

**Table 6:** Primer–probe pairs in the bovine-derived *cytb* gene.

F/R/P	Sequence (5′-3′)	Gene	Standards
F	CTCCTCGGAGACCCAGATAAC	*cytb*	SBT 10923-2012 [31]
R	AGAAGTATCACTCGGGTTTG		
P	FAM-CCAGCCAATCCACTCAACACACCC-TAMRA		

**Table 7:** Primer–probe pairs for the duck *IL-2* nuclear gene.

F/R/P	Sequence (5′-3′)	Gene
F	GGAGCACCTCTATCAGAGAAAGACA	*IL-2*
R	GTGTGTAGAGCTCAAGATCAATCCC	
P	FAM-TGGGAACAAAGCATGAATGTAAGTGGATGGT-BHQ1	

## Data Availability

The original contributions presented in this study are included in the article. Further inquiries can be directed to the corresponding authors.
